# Expanding the Regulon of the *Bradyrhizobium diazoefficiens* NnrR Transcription Factor: New Insights Into the Denitrification Pathway

**DOI:** 10.3389/fmicb.2019.01926

**Published:** 2019-08-20

**Authors:** Andrea Jiménez-Leiva, Juan J. Cabrera, Emilio Bueno, María J. Torres, Sergio Salazar, Eulogio J. Bedmar, María J. Delgado, Socorro Mesa

**Affiliations:** Department of Soil Microbiology and Symbiotic Systems, Estación Experimental del Zaidín, Consejo Superior de Investigaciones Científicas, Granada, Spain

**Keywords:** CRP/FNR proteins, *in vitro* transcription, microoxia, nitrogen oxides, Rhizobia, transcriptomics

## Abstract

Denitrification in the soybean endosymbiont *Bradyrhizobium diazoefficiens* is controlled by a complex regulatory network composed of two hierarchical cascades, FixLJ-FixK_2_-NnrR and RegSR-NifA. In the former cascade, the CRP/FNR-type transcription factors FixK_2_ and NnrR exert disparate control on expression of core denitrifying systems encoded by *napEDABC*, *nirK*, *norCBQD*, and *nosRZDFYLX* genes in response to microoxia and nitrogen oxides, respectively. To identify additional genes controlled by NnrR and involved in the denitrification process in *B. diazoefficiens*, we compared the transcriptional profile of an *nnrR* mutant with that of the wild type, both grown under anoxic denitrifying conditions. This approach revealed more than 170 genes were simultaneously induced in the wild type and under the positive control of NnrR. Among them, we found the *cycA* gene which codes for the *c*_550_ soluble cytochrome (CycA), previously identified as an intermediate electron donor between the *bc*_1_ complex and the denitrifying nitrite reductase NirK. Here, we demonstrated that CycA is also required for nitrous oxide reductase activity. However, mutation in *cycA* neither affected *nosZ* gene expression nor NosZ protein steady-state levels. Furthermore, *cycA*, *nnrR* and its proximal divergently oriented *nnrS* gene, are direct targets for FixK_2_ as determined by *in vitro* transcription activation assays. The dependence of *cycA* expression on FixK_2_ and NnrR in anoxic denitrifying conditions was validated at transcriptional level, determined by quantitative reverse transcription PCR, and at the level of protein by performing heme *c*-staining of soluble cytochromes. Thus, this study expands the regulon of NnrR and demonstrates the role of CycA in the activity of the nitrous oxide reductase, the key enzyme for nitrous oxide mitigation.

## Introduction

Denitrification is a form of bacterial respiration that occurs under oxygen limitation, in which the reduction of nitrate (NO_3_^–^) or nitrite (NO_2_^–^) to dinitrogen (N_2_) provides cellular energy. During this process, nitric oxide (NO) and nitrous oxide (N_2_O) are generated as free-intermediates (Zumft, 1997). Denitrification consists of four sequential enzymatic reactions catalyzed by nitrate-, nitrite-, nitric oxide-, and nitrous oxide reductases, encoded by *nar/nap, nir, nor*, and *nos* genes, respectively (reviewed in van Spanning et al., 2007; Kraft et al., 2011; Richardson, 2011; Torres et al., 2016). Denitrification is widely distributed within the domain of *Bacteria* and the majority of denitrifiers are found in the phylum *Proteobacteria* (Shapleigh, 2006). In this phylum, we found the soybean endosymbiont, *Bradyrhizobium diazoefficiens* (Delamuta et al., 2013), a soil Gram-negative bacterium that is also able to grow anoxically from nitrate respiration and to carry out the complete denitrification pathway. *B. diazoefficiens*, which is considered a model for studying rhizobial denitrification, possesses a periplasmic nitrate reductase (Nap), a copper-containing nitrite reductase (NirK), a *c*-type nitric oxide reductase (cNor), and a nitrous oxide reductase (Nos) encoded by *napEDABC*, *nirK*, *norCBQD*, and *nosRZDFYLX* genes, respectively (Velasco et al., 2001, 2004; Mesa et al., 2002; Delgado et al., 2003; reviewed in Bedmar et al., 2005, 2013).

In *B. diazoefficiens*, a complex regulatory network comprised by two coordinated cascades (FixLJ-FixK_2_-NnrR and RegSR-NifA) controls the expression of genes required for microoxic, symbiotic, and denitrifying lifestyles (reviewed in Sciotti et al., 2003; Fernández et al., 2016; Torres et al., 2016). In particular, the FixLJ-FixK_2_-NnrR cascade is activated at a concentration of ≤5% O_2_ at the level of the two-component regulatory system FixLJ, where the response regulator FixJ in its active phosphorylated form induces the expression of several genes, including *fixK_2_.* In turn, the FixK_2_ protein activates the expression of about three-hundred genes including those for denitrification and other regulatory genes such as *rpoN*_1_, *fixK*_1_, or *nnrR* (Mesa et al., 2008). The product of the latter, NnrR (nitrite and nitric oxide reductase regulator), is also required for maximal expression of key denitrification genes (Mesa et al., 2003; Bueno et al., 2017; Torres et al., 2017). Recently, distinct regulation of denitrifying gene expression in response to microoxia, different nitrogen oxides and the regulatory proteins FixK_2_ and NnrR was reported in *B. diazoefficiens*. In this regulatory cascade, expression of *napEDABC*, *nirK*, and *nosRZDFYLX* genes all require microoxic conditions and is directly dependent on FixK_2_ (Bueno et al., 2017; Torres et al., 2017), while NO is the key signal for the expression of *norCBQD*, being NnrR the regulator which directly interacts with the *norCBQD* promoter (Bueno et al., 2017).

Both FixK_2_ and NnrR proteins belong to the cyclic AMP receptor protein (CRP) and the fumarate and nitrate reductase (FNR) regulator superfamily of transcription factors that mainly act as activators in a wide range of bacteria (reviewed in Körner et al., 2003; Fernández et al., 2016; Torres et al., 2016). These proteins share four functional domains: an N-terminal sensor domain that usually binds a specific cofactor for functional activity, a β-barrel which interacts with the RNA polymerase, an α-helix involved in protein dimerization, and a C-terminal DNA-binding domain. Generally, the active form of this kind of transcription factor consists of a homodimeric protein complex which binds to a twofold symmetric consensus DNA sequence located at distinct distances in the promoters of the target genes. Specifically, in the case of FixK_2_, the consensus binding site is a 14 bp palindromic sequence, TTGA/C-N_6_-T/GCAA (Bonnet et al., 2013), based on the FixK_2_-DNA complex structure as well as the consensus deduced from the alignment of the FixK_2_ boxes present in the promoter region of not less than 14 FixK_2_ targets validated by *in vitro* transcription (IVT) activation assays (Mesa et al., 2008; Reutimann et al., 2010; Bueno et al., 2017). However, a single binding site has been already identified for NnrR which corresponds to the promoter region of the *norCBQD* genes that harbors a recognition sequence with high similarity to the consensus FixK_2_ box (Bueno et al., 2017).

The NnrR protein from *B. diazoefficiens* belongs to the wider NnrR clade of proteins that orchestrate the expression of the *nir* and *nor* gene clusters in order to keep the free concentrations of nitrite and NO below cytotoxic levels (Körner et al., 2003). Another subgroup in the CRP/FNR family is the DNR-type proteins such as Dnr from *Pseudomonas aeruginosa* which has a similar function as the NnrR-like proteins (Giardina et al., 2008). Some global transcriptomic studies have been performed previously to identify the regulons of Dnr from *P. aeruginosa* (Trunk et al., 2010), NnrR from *Rhodobacter sphaeroides* 2.4.1 (Arai et al., 2013), and DnrF from the marine bacterium *Dinoroseobacter shibae* (Ebert et al., 2017). However, such an approach has not been carried out for *B. diazoefficiens* NnrR.

In the present work, we expanded the NnrR regulon through a comparative transcriptomic analysis of *B. diazoefficiens* wild type (WT) and an *nnrR* mutant grown under anoxic denitrifying conditions. Among *nnrR* targets validated by quantitative reverse transcription PCR (qRT-PCR) analyses, we identified *cycA* that encodes the soluble cytochrome *c*_550_ (CycA). We also demonstrated the requirement of FixK_2_ for IVT of *cycA* and *nnrR* as well as the necessity of FixK_2_ and NnrR for maximal expression of *cycA in vivo*. Finally, we reveal the role of CycA in the activity of the nitrous oxide reductase, adding an extra function of this cytochrome in the denitrification pathway of *B. diazoefficiens*.

## Materials and Methods

### Bacterial Strains, Media, and Growth Conditions

Bacterial strains used in this study are listed in Supplementary Table S1 (all references cited in the Supplementary Material are compiled in Supplementary Data Sheet S1). *Escherichia coli* cells were routinely grown in Luria-Bertani (LB) medium (Miller, 1972) at 37°C. *E. coli* BL21 (DE3) cells for the overexpression of the recombinant FixK_2_ protein were incubated at 30°C. Where needed, antibiotics were used at the following concentrations (in μg/ml): ampicillin, 200; kanamycin, 30.

*B. diazoefficiens* cells were cultured oxically, microoxically (2% O_2_, 98% N_2_; headspace exchanged every 8–16 h) and anoxically (filled rubber-stoppered PYREX^®^ bottles without gas exchange) at 30°C, essentially as described previously (Torres et al., 2014; Bueno et al., 2017). While Peptone-Salts-Yeast extract (PSY) (Regensburger and Hennecke, 1983; Mesa et al., 2008) was the standard medium used for *B. diazoefficiens* culturing, Yeast Extract-Mannitol (YEM) medium supplemented or not with 10 mM KNO_3_ (Daniel and Appleby, 1972) was routinely employed in our experiments. In the latter case, cells were harvested by centrifugation (5,500 × *g* for 7 min at 4°C), washed twice with YEM medium and subsequently used for inoculation at an optical density at 600 nm (OD_600_) of 0.2. Cells were then incubated for 24 h at 30°C under rigorous shaking (170 rpm; oxic cultures) or gentle shaking (80 rpm; microoxic and anoxic cultures). The OD_600_ of cells at harvesting were: 0.75–0.85 (oxic conditions); 0.7–0.8 (microoxic conditions); 0.3–0.4 (anoxic conditions supplemented with nitrate, WT); 0.28–0.3 (anoxic conditions supplemented with nitrate, *fixK*_2_ and *nnrR* mutants). Antibiotics were added to cultures as required at the following concentrations (μg/ml): chloramphenicol, 20 (solid medium); streptomycin, 200 (solid medium), 100 (liquid medium); kanamycin, 200 (solid medium), 100 (liquid medium); spectinomycin, 200 (solid medium), 100 (liquid medium).

### Strains and Plasmids Construction

All strains and plasmids used in this work and their corresponding description are compiled in Supplementary Table S1. A *cy*_2_ (bll2388) deletion mutant was constructed by markerless mutagenesis using the *sacB*-system (Schäfer et al., 1994). For that purpose, the 5′ and 3′ flanking regions of the *cy*_2_ gene (614 and 609 bp fragments) were amplified by PCR using BLL1F/BLL1R and BLL2F/BLL2R primers pairs (Supplementary Table S2). Both fragments were individually cloned in the pGEM-T easy vector, verified by sequencing and finally cloned *in tandem* in the suicide vector pK18*mobsacB*, yielding plasmid pMB2003. This plasmid was transferred via biparental conjugation to *B. diazoefficiens* by using *E. coli* S17-1 as donor and transconjugants were firstly selected by kanamycin resistance (single recombination), and secondly by sucrose resistance (double recombination) as previously described (Cabrera et al., 2016). The genomic organization of selected candidates was verified by PCR using gene-specific primers. Double recombination of plasmid pMB2003 with the *B. diazoefficiens* genome resulted in the replacement of the wild-type *cy*_2_ gene encoding a 128 amino acids protein for an *in frame* truncated version encoding 23 amino acids in the *B. diazoefficiens cy*_2_ mutant (Supplementary Table S1).

Plasmids pMB1400 and pMB1401 were individually used as templates in the IVT activation assays. pMB1400 carries the divergent promoter of *nnrS* and *nnrR* genes on a *Bam*HI-*Eco*RI-digested 258-bp fragment amplified by PCR (combination of primers nnrR_6_for and nnrR_6_rev; Supplementary Table S2). The fragment was then cloned into plasmid pRJ8870 (Mesa et al., 2008), that harbors two *B. diazoefficiens rrn* transcriptional terminators. pMB1401 consists on an *Eco*RI-*Hin*dIII-digested 289-bp *cycA* promoter fragment that was amplified by PCR, using the cycA_1_for and cycA_1_rev primers (Supplementary Table S2) and then cloned into pRJ9519, which contains a *B. diazoefficiens rrn* transcriptional terminator (Beck et al., 1997).

The correct nucleotide sequence of all PCR-amplified fragments cloned in the corresponding plasmids was verified by sequencing.

### RNA Isolation, cDNA Synthesis, and Microarray Analysis

A custom-designed *B. diazoefficiens* GeneChip BJAPETHa520090 (Affymetrix, Santa Clara, CA, United States; Hauser et al., 2007) was used to analyze the transcriptional profile of the *nnrR* mutant in comparison to the WT. This GeneChip has successfully been used in a wealth of global transcriptional studies performed with *B. diazoefficiens* (reviewed in Lardi and Pessi, 2018). Both the *nnrR* mutant and WT strains were cultivated under anoxic conditions in YEM medium supplemented with nitrate (hereafter named as anoxic denitrifying conditions) as described above. After growth, cells were mixed with 0.1 volume of stop solution (10% buffered phenol in ethanol; Bernstein et al., 2002), and centrifuged at 9,000 rpm, 5 min, 4°C. After centrifugation, the pellet was immediately frozen in liquid nitrogen and stored at −80°C for a further use. For each strain, a minimum of three independent biological replicates were analyzed. RNA isolation, cDNA synthesis, fragmentation, and labeling, and conditions for microarray hybridization were done as described previously (Hauser et al., 2007; Lindemann et al., 2007; Pessi et al., 2007; Mesa et al., 2008; Torres et al., 2014). The details of data processing, normalization, and further analyses were described elsewhere (Pessi et al., 2007; Mesa et al., 2008; Torres et al., 2014). Only the probe sets that were called as “present” or “marginal” in 2 out of the 3 replicates were considered for further analyses. The group of differentially expressed genes comprised those that passed the statistical test (student *t*-test with a *p*-value threshold of 0.01) and also that their change in expression [determined as *n*-fold change (FC)] was ≥ 2 or ≤ − 2 in comparisons between two strains or two conditions. Prediction of putative operons was performed as described elsewhere (Hauser et al., 2007; Mesa et al., 2008).

### Gene Expression Determined by qRT-PCR

Expression of *norC*, *nosR*, *cycA, cy_2_*, and *rpoN*_1_ genes was also determined with qRT-PCR experiments using a QuantStudio 3 Real-Time PCR System (Thermo Fisher Scientific). Growth conditions of *B. diazoefficiens* WT and *nnrR* and *fixK*_2_ mutants, as well as RNA isolation and cDNA synthesis were performed as described above for the samples employed in the microarray experiments. All primers for qRT-PCR experiments are listed in Supplementary Table S2. Design of primers specifically used in this study was done with Clone Manager version 9 (Sci-Ed Software) to generate PCR fragments of about 100 bp and to have a melting temperature of 57°C to 62°C. For each target gene, qRT-PCR reactions were run in triplicate in a total volume of 19 μl containing 1 or 10 ng of cDNA, 2 μM (final concentration) of each primer and 9.5 μl of SYBR Green SuperMix (Bio-Rad). Relative changes in gene expression were calculated according to the methodology described by Pfaffl (2001). mRNA expression levels of each target gene were normalized to that of the sigma factor gene *sigA*.

### Detection of Soluble *c*-Type Cytochromes by Heme Staining

Three hundred milliliters of cultures of *B. diazoefficiens* strains grown oxically, microoxically, and in anoxic denitrifying conditions were harvested by centrifugation (5,000 × *g*, 7 min), washed with 50 mM Tris-HCl, pH 7.5 (fractionation buffer) and resuspended in one ml of the same buffer containing 1 mM 4-[2-Aminoethyl] benzenesulfonyl fluoride hydrochloride (AEBSF) and DNAse (20 μg/ml). Cell fractionation, sodium dodecyl sulfate gel electrophoresis (SDS-PAGE) and soluble cytochromes *c* staining were performed essentially as described elsewhere (Delgado et al., 2003; Mesa et al., 2008). Cells were disrupted by three passes through a cold French pressure cell (SLM Aminco, Jessup, MD, United States) at about 120 MPa, and subsequently centrifuged at 20,000 × *g* for 10 min at 4°C to remove the cells debris. Cell-free extracts were then ultracentrifuged at 140,000 × *g* for 45 min at 4°C and the resulted supernatant was mixed with one sixth volume of freshly prepared SDS loading dye (350 mM Tris-HCl, pH 6.8, 30% glycerol, 20 mM DTT, 350 mM SDS, 0.05% bromophenol blue). Soluble fractions (50 μg) were loaded without boiling onto 18%-SDS-PAGE polyacrylamide gels, subsequently resolved, and transferred to a nitrocellulose membrane using Trans-Blot Turbo System (Bio-Rad). Heme-dependent peroxidase activity of *c*-type cytochromes (Vargas et al., 1993) was detected using the ECL Select^TM^ Western Blotting Detection Reagent Kit (Amersham, GE Healthcare) in a ChemiDoc XRS + System. Image analyses were performed with the Quantity One and Image Lab^TM^ softwares (Bio-Rad). Experiments were done with at least two independent biological replicates.

### Immunoblot Detection of NosZ

Steady-state levels of NosZ protein were analyzed in the soluble fraction of cultures of *B. diazoefficiens* grown under microoxic conditions (2% O_2_) in YEM medium supplemented with nitrate. This growth condition was chosen to compare our results (also nitrous oxide reductase [N_2_OR] measurements; see below) with previous data described by Torres et al. (2017). At least two independent biological replicates of 25 ml of cells were harvested, washed with 40 mM Tris-HCl, 150 mM KCl, pH 7 (lysis buffer) and resuspended in one ml of the same buffer containing 1 mM AEBSF. Cell fractionation was performed as described above. Twenty microgram of protein was mixed with SDS loading dye (5:1 proportion, v/v), boiled and loaded onto SDS-14% polyacrylamide gels. Immunoblotting was carried out as previously described (Torres et al., 2017) using a heterologous antibody against NosZ of *Paracoccus denitrificans* (1:1,000 dilution) (Felgate et al., 2012). A horseradish peroxidase (HRP)-conjugated donkey anti-sheep antibody (Sigma-Aldrich) at a 1:3,500 dilution was used as secondary antibody. Immunoreactive proteins were detected and analyzed as described above for the heme-staining experiments.

### N_2_OR Activity

N_2_OR activity was measured as N_2_O consumption in *B. diazoefficiens* cultures grown microoxically in the presence of nitrate. 200 ml of cells were harvested by centrifugation (5,000 × *g*, 7 min) and washed at least four times with 50 mM Tris-HCl, pH 7.5, to remove the remaining nitrate and nitrite. Cell pellets were then resuspended in 4 ml of the same buffer prior to N_2_OR activity determination in the absence (endogenous activity) or in the presence of succinate. Reaction mixtures were individually carried out in 17 ml rubber-stoppered tubes which contained 7 ml of 50 mM Tris-HCl, pH 7.5, cell suspension (0.8–1 mg of protein), and 700 μl of a solution of 600 mM succinate, where needed. 500 μl of the headspace was exchanged by a gas mixture containing 2% N_2_O in 98% N_2_, and incubated for 30 min prior to adding cell suspension to ensure equilibrium between headspace and liquid. Four hours after the reactions were set up, aliquots of one ml of the headspace were taken and analyzed for N_2_O consumption essentially as described previously (Tortosa et al., 2015; Torres et al., 2017). N_2_O was measured by gas chromatography with a PorapaK Q 80/100 MESH column and N_2_ as carrier gas at 23 ml/min. The nmoles of N_2_O consumed were calculated based on a calibration curve done with different volumes of N_2_O. Activity was expressed as nmol N_2_O consumed per (mg prot)^–1^h^–1^.

### IVT Activation Assay

Multiple-round IVT activation assays were essentially performed as described earlier (Beck et al., 1997; Mesa et al., 2005, 2008; Torres et al., 2017). Briefly, transcription reactions were carried out in a 50 μl reaction volume containing transcription buffer (40 mM Tris-HCl pH 8, 10 mM MgCl_2_, 0.1 mM EDTA, 0.1 mM DTT, 150 mM KCl, 0.4 mM K_3_PO_4_), 20 U of RNAse inhibitor (Roche), 1 mM of each NTP, 1 μCi [alpha-^32^P] UTP (800 Ci mmol; PerkinElmer), 750 ng of the corresponding DNA template (pMB1401 or pMB1400) and 1.5 μg of RNA polymerase of *B. diazoefficiens* purified as described previously (Torres et al., 2017). A C-terminally His-tagged C183S FixK_2_ protein (insensitive to oxidation; hereafter named as FixK_2_) (Bonnet et al., 2013) at 1.25 and 2.5 μM was added to the reaction mixtures where needed. Reactions were incubated 30 min at 37°C, placed on ice to stop the reaction, treated with phenol:chloroform:isoamyl alcohol and incubated overnight at −20°C to precipitate RNA. RNA size markers were prepared with T3 RNA polymerase as described elsewhere (Mesa et al., 2005). Samples were resuspended in Sanger solution (95% formamide, 40 mM EDTA pH 8, 0.05% bromophenol blue, 0.05% xylene cyanol), heated at 90°C for 5 min, centrifuged at 13,000 × *g* for 1 min and loaded onto a 6% polyacrylamide-7 M urea gel, which run at a constant power of 15 W. For the visualization of the radioactive signal transcripts, vacuum dried-gels were exposed to a phosphoimager screen (Molecular Dynamics) for at least 3 days. The images were analyzed with the Image Lab^TM^ software (Bio-Rad).

### Measurement of Protein Concentration

Protein concentration was measured using the Protein assay Dye Reagent Concentrate (Bio-Rad Laboratories) with bovine serum albumin (BSA) as standard protein for the calibration curve.

## Results

### Global Transcriptional Profiling of the *nnrR* Mutant Cultured in Anoxic Denitrifying Conditions

**FIGURE 1:**
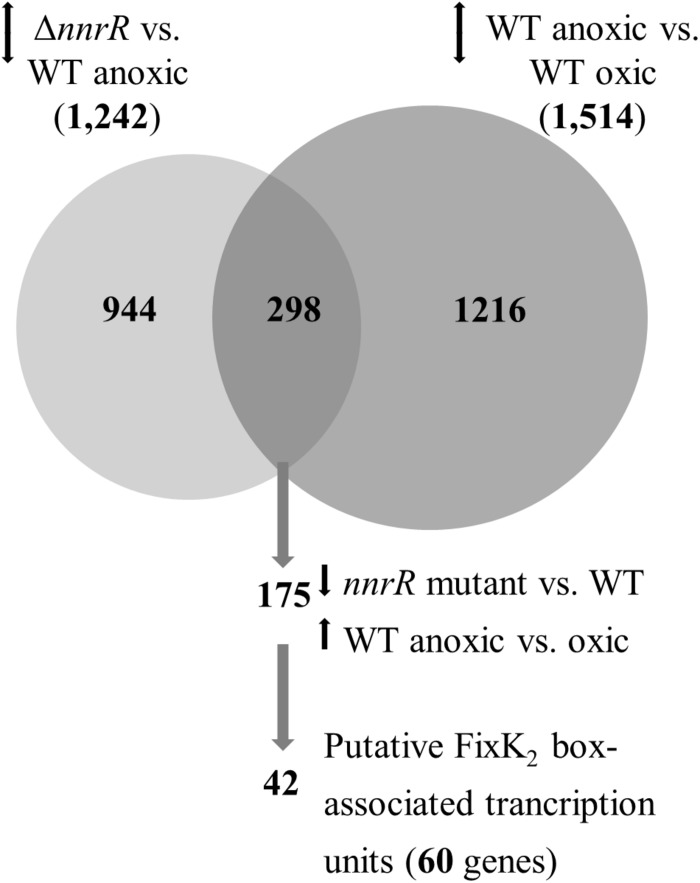
Venn diagram representing the group of differentially expressed genes in the *nnrR* mutant in comparison to the WT, both grown in anoxic denitrifying conditions (light gray circle), and the group of differentially expressed genes in cells of the WT grown in anoxic denitrifying conditions compared with cells grown in oxic conditions (dark gray circle). Numbers in parentheses indicate the total number of differentially expressed genes. The overlap of both groups represents 298 genes; among them, 175 genes showed a downregulated expression in the *nnrR* mutant and at the same time are induced in the WT grown in anoxic denitrifying conditions. The latter set contains 60 genes organized in mono-, or polycistronic transcriptional units that contain a putative FixK_2_ box in the promoter region (42 FixK_2_ boxes associated to transcriptional units, see Table 1). Up-down arrows refer to increased and decreased gene expression in microarray analyses.

**FIGURE 2:**
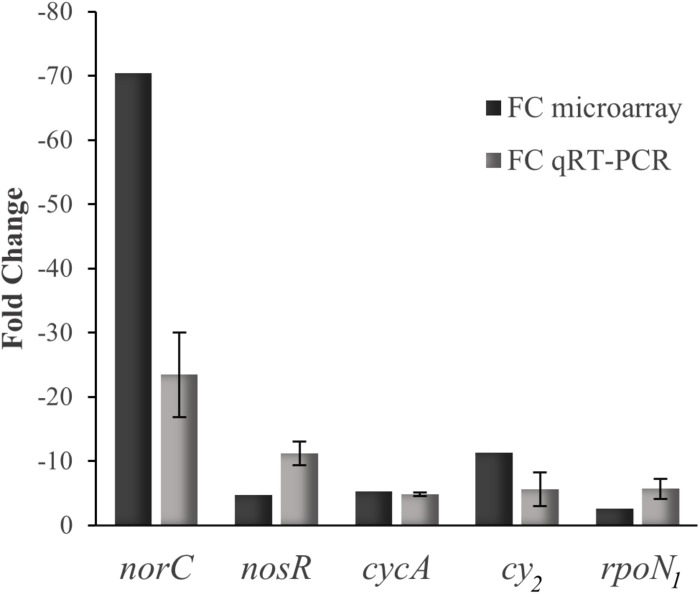
Expression data of selected genes determined by microarray (black bars) and qRT-PCR (gray bars). Fold change (FC) refers to the relative expression of *norC*, *nosR*, *cycA*, *cy_2_, norC*, and *rpoN*_1_ genes in the *nnrR* mutant in comparison with the WT, both cultured in anoxic denitrifying conditions. qRT-PCRs were performed with two independent experiments, each one with six parallel amplification reactions.

The comparative transcriptional profile of a *B. diazoefficiens nnrR* mutant relative to WT, during anoxic denitrifying conditions and using a custom-made GeneChip array (Hauser et al., 2007), revealed 1,242 genes that showed a differential expression in the *nnrR* mutant (Figure 1). The overlap of this group with those genes differentially controlled in wild-type cells cultivated in anoxic denitrifying conditions in comparison to oxically cultivated cells (1,514 genes) resulted in 298 genes (Figure 1). We next focused on those, among this latter group, which were induced in the WT and, at the same time, showed a downregulated expression in the *nnrR* mutant (i.e., NnrR exerts its role as an activator). The comparison of both regulons yielded 175 genes (Figure 1 and Supplementary Table S3). Next, in order to narrow down the number of genes for further analysis, we did a systematic search for promoter-associated putative FixK_2_ binding sites as a first approximation, since no consensus sequence for NnrR binding has been defined. Due to the homology between the consensus FixK_2_ box (Bonnet et al., 2013), and the only so far known NnrR binding site present in the *norCBQD* promoter (Bueno et al., 2017), it can argued that the FixK_2_ box might also serve as recognition site also for NnrR. From this, we then identified 60 genes within the 175 genes group that form part of 42 predicted transcriptional units (mono- or poly-cistronic) associated with a putative FixK_2_ box (**Table 1**). Within this group we found: (i) Three of the four structural gene clusters involved in denitrification, i.e., *nirK* (Velasco et al., 2001), *norCBQD* (Mesa et al., 2002) and *nosRZDFYLX* (Velasco et al., 2004), encoding the NirK, cNor, and Nos enzymes, respectively; (ii) genes encoding cytochromes, such as bll2388 (*cy*_2_) (Mesa et al., 2008), and *cycA* (cytochrome *c*_550_) (Bott et al., 1995); (iii) regulatory genes as *nnrR* itself and *rpoN*_1_ that encodes one of the two copies of the alternative sigma factor RpoN that collaborates with the transcription factor NifA for activation of genes involved in nitrogen fixation (reviewed in Dixon and Kahn, 2004). The downregulation of the *nnrR* gene observed in the *nnrR* mutant indicates, as it was described previously by Mesa et al. (2003), that NnrR does not negatively auto-regulate its own expression; (iv) two copies of the oxygen-independent coproporphyrinogen-III oxidase involved in heme biosynthesis under oxygen limitation (i.e., *hemN*_1_ and *hemN*_2_); (v) the *phbB* gene encoding PhaB, one of the two copies of NADP–acetoacetyl-CoA reductases involved in the second step of polyhydroxybutyrate (PHB) biosynthesis (Quelas et al., 2013). We next selected few candidates listed in Table 1 for validation by qRT-PCR of their gene expression profile based on microarray data. In these experiments, we could confirm that *norC, nosR, cycA*, *cy_2_*, and *rpoN*_1_ genes are targets positively activated by NnrR (Figure 2), being *cycA* and its product the focus of our study.

**TABLE 1:** List of the 60 genes belonging to 42 putative FixK_2_ box-associated transcription units whose expression is downregulated in the *nnrR* mutant in comparison to the WT, both cultured under anoxic denitrifying conditions, and at the same time are induced in the WT in anoxic denitrifying conditions compared to oxic conditions.

**Query^a^**	**FC (Δ*nnrR* anoxia vs. WT anoxia)^b^**	**FC (WT anoxia vs. WT oxia)^c^**	**Locus_tag^d^**	**Gene name^e^**	**Product^f^**	**Position^g^**	**Motif^h^**	**Predicted operon structure^i^**
bll0225	–2.7	3.1	Bdiaspc4_00775	*phbB*	Acetoacetyl-CoA reductase	–86	TTGATGTCCGTCAA	–
blr0314	–4.8	23.1	Bdiaspc4_01230	*nosR*	Regulatory protein NosR	–131	TTGATCCAGCGCAA	blr0314-blr0320
blr0315	–6.4	25.7	Bdiaspc4_01235	*nosZ*	TAT-dependent nitrous-oxide reductase	–	–	–
blr0316	–6.4	15.4	Bdiaspc4_01240	*nosD*	Nitrous oxide reductase family maturation protein NosD	–	–	–
blr0317	–7.5	13.7	Bdiaspc4_01245	*nosF*	ABC transporter ATP-binding protein	–	–	–
blr0318	–9.3	23.1	Bdiaspc4_01250	*nosY*	ABC transporter permease	–	–	–
blr0319	–6.2	10.2	Bdiaspc4_01255	*nosL*	Copper resistance protein CopZ	–	-	–
blr0320	–6.0	13.1	Bdiaspc4_01260	*nosX*	FAD:protein FMN transferase	–	–	–
blr0964	–4.3	2.0	Bdiaspc4_04660	–	NAD(P)/FAD-dependent oxidoreductase	–41	TTGATCTATGTCAT	–
blr0984	–5.6	2.4	Bdiaspc4_04770	–	AraC family transcriptional regulator	–245	TTGCTGCGGAGCAG	–
blr1311	–7.6	52.2	Bdiaspc4_06505	–	OmpW family protein	–60	TTGATCGGCGTCAA	–
bll1766	–7.1	19.6	Bdiaspc4_08945	–	OmpW family protein	–228	TTGATTGGTATCAA	–
blr1883	–2.6	9.5	Bdiaspc4_09550	*rpoN*_1_	RNA polymerase σ-^54^ factor	–81	TTGCGCGACATCAA	–
bll1944	–3.7	8.5	Bdiaspc4_09875	–	Porin family protein	–174	TGGAGCGACACCAA	–
blr2006	–2.3	2.1	Bdiaspc4_10180	*id676*	Hypothetical protein	–451	TTGATCAGGTGCAA	–
bll2007	–8.3	56.9	Bdiaspc4_10185	*hemN*_1_	Oxygen-independent coproporphyrinogen III oxidase	–138	TTGACATAACGCAA	–
bll2330	–4.2	21.6	Bdiaspc4_11930	–	VOC family protein	–73	TTGATCCAGATCAA	bll2330-bll2329-bsl2328
bll2329	–3.7	7.2	Bdiaspc4_11925	–	FAD-dependent oxidoreductase	–	–	–
bsl2328	–3.5	6.6	Bdiaspc4_11920	–	DUF2783 domain-containing protein	–	–	–
bll2388	–11.3	15.2	Bdiaspc4_12230	*cy*_2_	Cytochrome *c* family protein	–435	TTGCGCCGGATCAG	–
bll2664	–2.3	3.9	Bdiaspc4_13755	–	DUF1254 domain-containing protein	–384	CTGCTCGACCTCAA	bll2664-bll2663
blr2763	–2.1	58.7	Bdiaspc4_14290	*ccoN/fixN*	Cytochrome-*c* oxidase, *cbb*_3_-type subunit I	–70	TTGATTTCAATCAA	blr2763-blr2764-bsr2765-blr2766
blr2764	–2.9	55.5	Bdiaspc4_14295	*ccoO/fixO*	Cytochrome-*c* oxidase, *cbb*_3_-type subunit II	–	–	–
blr2932	–7.6	7.3	Bdiaspc4_15155	–	Methyl-accepting chemotaxis protein	–124	TCGAGCCGGCTCAA	–
blr2933	–6.2	2.8	Bdiaspc4_15160	–	PAS domain S-box protein	–294	TCGGGCCGGCTCAA	–
bsr3073	–3.8	4.2	Bdiaspc4_15875	–	Hypothetical protein	–101	TTGACGCGGATCAA	–
bsl3211	–35.6	36.5	Bdiaspc4_16610	–	Hypothetical protein	–411	TTGATCGCGATGAA	–
blr3212	–105.4	53.8	Bdiaspc4_16615	*norE*	Cytochrome *c* oxidase subunit 3 family protein	–106	TTGCGTCGGCGCAA	blr3212-bsr3213
bsr3213	–14.6	7.5	Bdiaspc4_16620	–	Hypothetical protein	–	–	–
blr3214	–70.4	81.2	Bdiaspc4_16625	*norC*	Cytochrome *c*	–87	TTGCGCCCTGACAA	blr3214-blr3217
blr3215	–159.2	71.2	Bdiaspc4_16630	*norB*	Nitric oxide reductase subunit B	–	–	–
blr3216	–69.9	56.3	Bdiaspc4_16635	*norQ*	CbbQ/NirQ/NorQ/GpvN family protein	–	–	–
blr3217	–16.8	17.6	Bdiaspc4_16640	*norD*	VWA domain-containing protein	–	–	–
bll3611	–4.3	2.3	Bdiaspc4_18640	–	Caspase family protein	–330	TTGAACCACGTCAG	–
bll3835	–6.0	31.6	Bdiaspc4_19825	–	PepSY domain-containing protein	–93	TTGCTGCAAATCAA	–
blr4191	–6.7	6.0	Bdiaspc4_21720	–	Histidine kinase	–187	TTGATCTGGATCAA	–
blr4352	–7.5	3.2	Bdiaspc4_22595	–	Porin family protein	–433	TTGCGGGCGTGCAA	–
bsl4623	–3.4	4.9	Bdiaspc4_24190	–	Hypothetical protein	–71	TTGATGAAGATCAA	–
blr4770	–2.8	2.3	Bdiaspc4_25015	–	Lytic transglycosylase domain-containing protein	–246	TTGCGTCGGATCGA	–
bll5026	–7.0	2.3	Bdiaspc4_26405	*hppa*	K + -insensitive pyrophosphate-energized proton pump	–83	TTGTTCGAAATCAA	–
blr5774	–2.7	11.7	Bdiaspc4_30495	–	NAD(P)/FAD-dependent oxidoreductase	–459	TTGATCTTGCTCAA	blr5774-blr5775-bsr5776
blr5775	–4.1	10.8	Bdiaspc4_30500	*trxC*	Thioredoxin TrxC	–	–	–
bsr5776	–2.7	26.2	Bdiaspc4_30505	–	DUF2892 domain-containing protein	–	–	–
bll5842	–3.3	2.3	Bdiaspc4_30840	*flaF*	Flagellar biosynthesis regulatory protein FlaF	–43	TTAAGCGCGTTCAA	–
bll6222	–17.1	17.8	Bdiaspc4_32820	–	Group III truncated hemoglobin	–89	TTGCGCTGCGACAA	–
blr6437	–5.7	2.1	Bdiaspc4_33940	–	SMP-30/gluconolactonase/LRE family protein	–80	TTGACAGGTCTCAA	–
bll6496	–4.0	2.1	Bdiaspc4_34245	–	EALdomain-containing protein	–374	ATGCCCTGGATCAA	–
blr7084	–4.1	4.4	Bdiaspc4_37380	*nnrR*	CRP/FNR family transcriptional regulator	–66	TTGCGCTATCGCAA	–
bsl7085	–29.2	133.8	Bdiaspc4_37385	–	DUF1858 domain-containing protein	–62	TTGCGCTCCAACAA	–
bll7086	–4.3	43.8	Bdiaspc4_37390	*hemN*_2_	Oxygen-independent coproporphyrinogen III oxidase	–140	TTGCGCGAGCGCAA	–
blr7089	–21	161.4	Bdiaspc4_37405	*nirK*	Nitrite reductase, copper-containing	–74	TTGTTGCAGCGCAA	–
blr7544	–5.3	2.2	Bdiaspc4_39800	*cycA*	Cytochrome *c* family protein	–137	TTGTTGCAGCGCAA	–
bll7628	–10.3	10.1	Bdiaspc4_40255	–	Sterol-binding protein	–48	TTGTTCCCGCTCAA	bll7628-bll7627
bll7627	–15	75.3	Bdiaspc4_40250	–	U32 family peptidase	–	–	–
blr7684	–4.0	2.7	Bdiaspc4_40605	–	Hypothetical protein	–399	TTGATGTAGGTCGA	blr7684-blr7685
blr7685	–3.4	2.4	Bdiaspc4_40610	–	PilZ domain-containing protein	–	–	–
bll7787	–3.1	23.0	Bdiaspc4_41200	–	Hypothetical protein	–118	TTGACCCAGATCAA	–
blr7961	–3.3	43.7	Bdiaspc4_42105	–	Hsp20/alpha crystallin family protein	–82	TTGAGACAAATCAA	–
bll7982	–4.3	31.2	Bdiaspc4_42210	–	Class I SAM-dependent methyltransferase	–96	TTGATCTGAAACAA	bll7982-bll7981
bll7981	–5.9	22.1	Bdiaspc4_42205	–	Dehydrogenase	–	–	–

*^*a*^Best blast hit in the B. diazoefficiens USDA 110 genome (Kaneko et al., 2002; GenBank acc. # NC_004463.1; RefSeq annotation as from January 2016). ^*b*^Fold change (FC) values of gene expression in the nnrR mutant in comparison to the WT, both grown in anoxic dentrifying conditions. ^*c*^FC values of gene expression in wild-type cells grown under anoxic denitrifying conditions in comparison to those in cells grown oxically. ^*d*^Nomenclature of B. diazoefficiens 110spc4 genes according to the NCBI annotation (GenBank acc. # CP032617; Fernández et al., 2019). ^*e*^Gene name according to the NCBI annotation with modifications shaded in gray (GenBank acc. # CP032617; Fernández et al., 2019). ^*f*^Protein/gene product according to the NCBI annotation (GenBank acc. # CP032617; Fernández et al., 2019). ^*g*^Position of the first nucleotide of the motif relative to the annotated translational start site of the associated gene. ^*h*^Predicted putative FixK_2_ binding site. ^*i*^Prediction of the operon structure as described in Materials and Methods and previous published data.*

### NnrR and FixK_2_ Are Required for the Anoxic Induction of CycA

**FIGURE 3:**
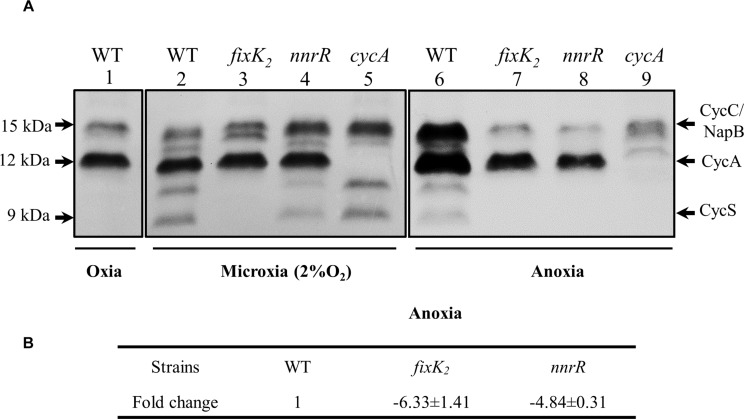
The regulatory proteins FixK_2_ and NnrR are involved in the control of *cycA* expression. **(A)** Profile of heme-stained soluble proteins from *B. diazoefficiens* WT, and *fixK*_2_, *nnrR*, and *cycA* mutants. Cells were cultivated during 24 h in YEM medium in the absence (oxic, and microoxic [2% O_2_] conditions) or in the presence (anoxic denitrifying conditions) of 10 mM of KNO_3_. 50 μg of soluble proteins were loaded per lane. Heme stained *c*-type cytochromes identified previously are specified at the right margin and their predicted molecular mass at the left. Each panel corresponds to different sections of the same gel (soluble fractions isolated from oxically and microoxically grown cells; Supplementary Data Sheet S2A) or a different gel (soluble fractions of cells grown in anoxic denitrifying conditions; Supplementary Data Sheet S2A. **(B)** qRT-PCR analyses of *cycA* in the WT, and *fixK*_2_ and *nnrR* mutants. Six parallel reactions were performed with cDNA retro-transcribed from RNA isolated from cells grown in anoxic denitrifying conditions (at least two independent biological replicates per strain). Fold change (FC) values refer to the relative expression in the *fixK*_2_ and *nnrR* mutants compared to the WT. anoxia, anoxic denitrifying conditions.

The *cycA* gene codes for the so-called cytochrome *c*_550__,_ a *c*-type soluble cytochrome (CycA) (Bott et al., 1995), previously identified as an intermediate electron donor between the cytochrome *bc*_1_ membrane complex and the denitrifying nitrite reductase NirK (Bueno et al., 2008). In order to confirm the positive control of NnrR on *cycA* also at protein level, we monitored the expression of CycA in the WT, and *nnrR* mutant by using heme-*c* staining analyses (Figure 3A). A *cycA* mutant was included as control in the experiments. For this purpose, the soluble fraction was isolated from cells grown under oxic, microoxic and anoxic denitrifying conditions (see Materials and Methods for further details). As shown in Figure 3A (lanes 1, 2 and 6), two stained bands of about 15 and 12 kDa were observed in the soluble fraction of wild-type cells independently of the growth conditions. The 15 kDa band corresponds to the previously identified cytochrome *c*_555_ (CycC) encoded by *cycC* (Bott et al., 1995), and the NapB subunit of the periplasmic nitrate reductase (Delgado et al., 2003) that co-migrate together. The 12 kDa-band was not detected in the *cycA* mutant (Figure 3A, lanes 5, and 9), indicating that this band corresponds to the CycA holoprotein (processed with covalently bound heme) with a predicted molecular mass of 12.320 kDa. Similarly, Bott et al. (1995) identified the 12 kDa band in the heme *c-*staining profiling of soluble cytochromes detected in oxic conditions as cytochrome *c*_550_ (CycA).

As shown in Figure 3A (lanes 1 and 2), CycA levels were similar in oxic or microoxic cultures of wild-type cells, while an about twofold induction of the CycA band was observed in the soluble fraction of the WT grown under anoxic denitrifying conditions (Figure 3A, comparison of lane 6 and lanes 1, and 2). In addition to the 15- and 12- kDa bands, two smaller cytochromes of about 10 and 9 kDa were also detected in the soluble fraction of wild-type cells cultured microoxically or in anoxic denitrifying conditions (Figure 3A, lanes 2, and 6). While the 10 kDa *c*-type cytochrome has not been yet identified, the 9 kDa *c*-type cytochrome corresponds to the soluble cytochrome CycS expressed in anaerobic, nitrate-grown cells (Mesa et al., 2008).

In the pattern of soluble *c*-type cytochromes detected in the *nnrR* mutant (Figure 3A, comparison of lanes 4 and 2), CycA expression was similar to that detected in wild-type cells when both strains were grown microoxically. However, under anoxic denitrifying conditions, the levels of CycA in the *nnrR* mutant were about two to three-fold lower to that of the WT (Figure 3A, lanes 6, and 8). Next, given the dependency of *nnrR* expression on the transcription factor FixK_2_ (Mesa et al., 2003, 2008), we also analyzed the expression of CycA in a *fixK*_2_ mutant cultured under microoxic and anoxic denitrifying conditions. Interestingly, we detected a similar expression profile for CycA in the *fixK*_2_ mutant to the one observed in the *nnrR* mutant (Figure 3A, lanes 3, and 7), which is consistent with the requirement of both FixK_2_ and NnrR for the maximal expression of CycA. This mutual dependence of CycA expression on these two CRP/FNR transcription factors was also confirmed at transcriptional levels by monitoring *cycA* expression by qRT-PCR (Figure 3B) which was about six and fivefold lower in the *fixK*_2_ and *nnrR* mutants, respectively, compared to the WT.

### FixK_2_ Directly Controls *cycA*, *nnrR*, and *nnrS* Genes Transcription

**FIGURE 4:**
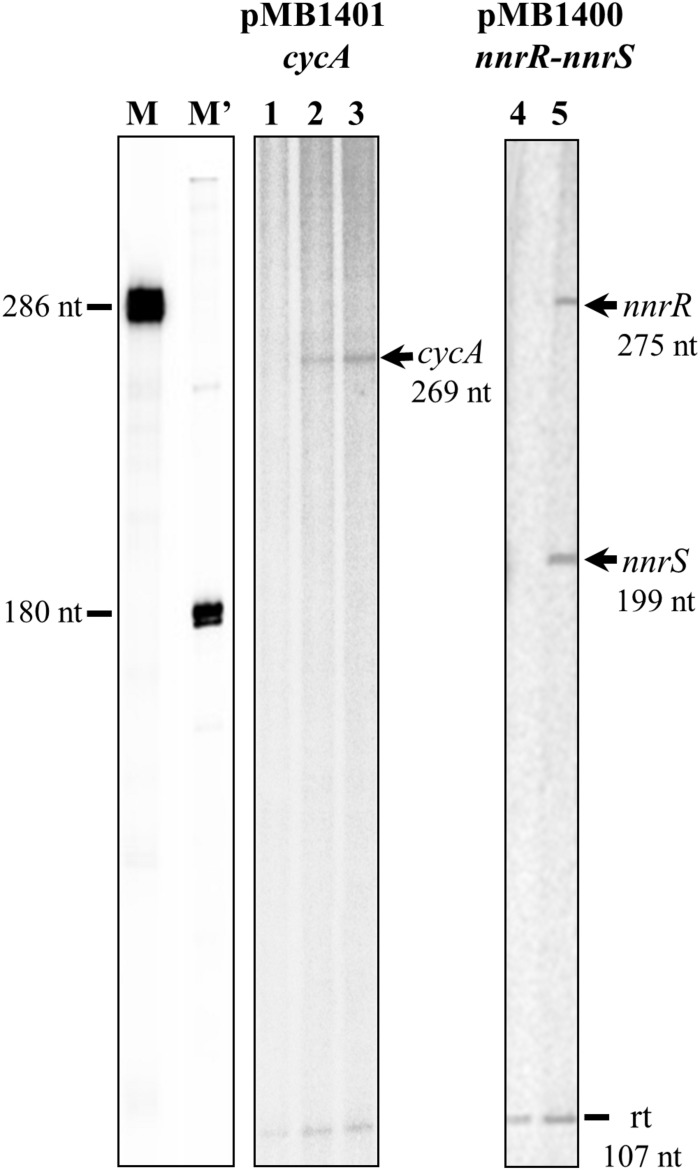
IVT activation of *nnrR*, *nnrS* and *cycA* genes by FixK_2_. Plasmids pMB1400 and pMB1401 were used for a multiple-round IVT activation assay with purified FixK_2_ protein (no protein, lanes 1, and 4; 1.25 μM, lane 2, and 5; 2.5 μM, lane 3) and RNA polymerase from *B. diazoefficiens*. RNA size markers (M, M′) were produced as described earlier (Mesa et al., 2005). The positions of the FixK_2_-dependent transcripts are marked with arrows. A vector-encoded transcript of 107 nucleotides (reference transcript) which serves as internal control appears in all lanes. Transcription products generated from each promoter were run on different gels. nt, nucleotides; rt, reference transcript.

Multiple-round IVT activation assays were performed to investigate whether *cycA* is directly controlled by the transcriptional regulator FixK_2_. To achieve this goal, we monitored transcription from the *cycA* promoter, cloned in plasmid pMB1401, with the native RNA polymerase purified from *B. diazoefficiens* (Torres et al., 2017) in the presence or in the absence of recombinant FixK_2_ protein. Without FixK_2_, *B. diazoefficiens* RNAP failed to transcribe from the *cycA* promoter (Figure 4, lane 1) whereas it produced a vector-encoded transcript of 107 nucleotides that it used as an internal control. In the presence of two different concentrations of FixK_2_ (1.25 and 2.5 μM dimer), *B. diazoefficiens* RNAP transcribed the *cycA* promoter efficiently, producing an expectable transcript of 269 nucleotides, with almost no change in the intensity of the 107-nucleotide control (Figure 4, lanes 2 and 3).

Previous work has shown that the activation of a *nnrR*′-′*lacZ* fusion under anoxic denitrifying conditions depends on FixK_2_ (Mesa et al., 2003). In order to elucidate whether FixK_2_ exerts direct or indirect control on *nnrR*, we also performed IVT analyses from the *nnrR* promoter. *nnrR* and its divergent oriented gene *nnrS* share a single predicted FixK_2_ box located at their intergenic promoter region which was cloned into the template plasmid pRJ8870 (Mesa et al., 2008), that carries two transcriptional terminators. As shown in Figure 4 (lane 5), *B. diazoefficiens* RNAP transcribed the *nnrS* and *nnrR* promoters only in the presence of purified FixK_2_ protein producing two expectable transcripts of 275 nucleotides (corresponding to *nnrR*), and 199 nucleotides (corresponding to *nnrS*). Collectively, these results allowed us to identify three new direct targets for FixK_2_: *cycA*, *nnrR*, and *nnrS*.

### CycA Is Involved in Nitrous Oxide Reductase Activity

**FIGURE 5:**
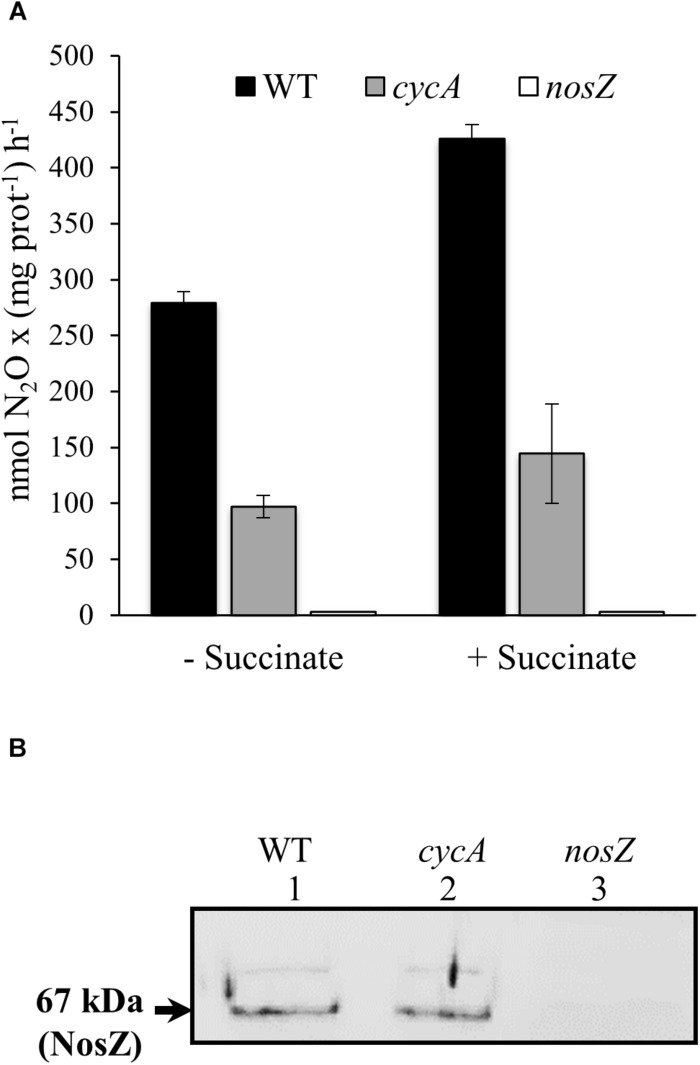
CycA is involved in nitrous oxide reductase activity. **(A)** N_2_OR activity determined in the WT and *cycA* mutant in the absence or in the presence of succinate as physiological electron donor. Activity is expressed as nmol N_2_O consumed per (mg prot)^–1^h^– 1^. Shown are means with standard errors of the three measurements of one representative experiment from at least two independent biological replicates. **(B)** Immunodetection of NosZ protein in the soluble fraction of the WT and *cycA* mutant. 20 μg of the soluble fraction of each strain were resolved by SDS-PAGE and immunoblotted with anti-NosZ antibody from *P. denitrificans* (for a detailed description see section Materials and Methods). The size of the predicted molecular mass of the mature periplasmic NosZ protein (67 kDa) is indicated on the left side. The original image of the immunoblot is shown in Supplementary Data Sheet S2B. In both series of experiments **(A,B)**, cells were grown microoxically (2% O_2_) for 24 h in YEM medium supplemented with nitrate. A *nosZ* mutant was included as negative control.

In order to investigate whether CycA could act as a potential electron donor to the nitrous oxide reductase (N_2_OR) enzyme, we determined N_2_OR activity in wild-type and *cycA* mutant cells under microoxic conditions (2% O_2_) in YEM medium supplemented with nitrate. Cells were cultured for 24 h, harvested by centrifugation and subsequently incubated microoxically in a reaction mixture supplemented with or without succinate as physiological electron donor. The ability of the cells to consume N_2_O was measured at 2 and 4 h after cell inoculation. As shown in Figure 5A, despite of the fact that the presence of succinate in the medium increased about 1.5-fold N_2_OR activity in both WT and *cycA* mutant, values of N_2_OR activity in *cycA* cells were about 65% lower than those observed in wild-type cells. As expected, almost no N_2_OR activity was detected in cells of a *nosZ* mutant (Figure 5A).

We also analyzed the expression of *nosR*, the first gene of the *nosRZDFYLX* operon, by qRT-PCR as well as the steady-state levels of NosZ, the catalytic subunit of N_2_OR in the WT and the *cycA* mutant. qRT-PCR analyses showed that *nosR* expression was not significantly affected in the *cycA* mutant (FC of −2.9) compared to wild-type cells, both cultivated under microoxic conditions with nitrate. Western blot analysis of NosZ in the soluble fraction of the *cycA* mutant and wild-type cells using an antibody against purified *P. denitrificans* NosZ (Felgate et al., 2012) showed that a band of about 67 kDa corresponding to the predicted molecular mass of the mature periplasmic NosZ protein, was detected with a similar intensity in both strains (Figure 5B, lanes 1, and 2). This band was absent in the soluble fraction extracted from *B. diazoefficiens nosZ* mutant cells which confirmed that corresponds to NosZ (Figure 5B, lane 3).

## Discussion

Denitrification in bacteria (reviewed in Zumft, 1997; van Spanning et al., 2007; Kraft et al., 2011; Richardson, 2011; Bueno et al., 2012; Torres et al., 2016), and specifically in the model organism *B. diazoefficiens* (reviewed in Bedmar et al., 2005, 2013) has been widely studied to date. The regulatory protein NnrR plays an important role in *B. diazoefficiens* since it expands the regulatory cascade FixLJ-FixK_2_ and integrates the nitrogen oxide signal (Mesa et al., 2003). However, the regulon of NnrR has not been yet fully mapped. In this work, a transcriptomic analysis of an *nnrR* mutant in comparison to the WT, both cultured under anoxic denitrifying conditions, was performed and compared with the group of anoxically-induced genes in the WT. This comparison led the identification of 175 genes induced by anoxia under the positive regulatory control of NnrR (Supplementary Table S3). Since the change in expression of some of these genes might be due to indirect effects (e.g., different grow behavior of the *nnrR* mutant and the WT, or the putative involvement of another regulatory protein), we focused on a group of 60 genes that contains a FixK_2_ binding site within their promoter regions (Table 1). Among this group, three targets (*nirK*, *norCBQD*, *nosRZDFYLX*) were previously reported to be under its direct or indirect control (Mesa et al., 2003; Robles et al., 2006; Bueno et al., 2017; Torres et al., 2017). Particularly, our results confirm those that showed a reduced expression of *nirK-lacZ*, *norC-lacZ*, or *nosR-lacZ* fusions in an *nnrR* mutant (Bueno et al., 2017; Torres et al., 2017). However, the only known direct target for NnrR is *norCBQD* gene cluster, since purified recombinant NnrR protein was able to bind its promoter region in an anoxic-specific manner (Bueno et al., 2017), while the downregulation of *nirK*, and *nosRZDFYLX* was attributed to the toxic effect of NO accumulation in the *nnrR* mutant (Torres et al., 2017). In this work, we also identified as NnrR targets genes involved in electron transport through the denitrification pathway. This is the case of *cycA* encoding a cytochrome *c*_550_ which acts as an electron donor to NirK (Bueno et al., 2008), and *cy*_2_ that encodes cytochrome *c*_2_, which was previously described as an indirect FixK_2_ target (Mesa et al., 2008). Other genes putatively activated by NnrR are *hemN*_1_ and *hemN*_2_, where the latter encodes a gene product required for denitrification as well as for symbiotic nitrogen fixation as reported by Fischer et al. (2001). In addition to the genes described above, we also found *rpoN*_1_ as a target under the positive control of NnrR which points to additional cross-control between the two oxygen-responsive regulatory cascades RegSR-NifA and FixLJ-FixK_2_-NnrR. In fact, 14 out the 65 genes that constitute the direct NifA + RpoN_1+2_ regulon (Hauser et al., 2007) showed a downregulated expression in the *nnrR* mutant (Supplementary Table S3). Taken together, these results expand the *B. diazoefficiens* NnrR regulon under denitrifying conditions. In other rhizobia such as *Ensifer meliloti*, transcriptomic studies were consistent with our findings, and identified denitrification genes (*nirK* and *norC*), as well as other genes associated to denitrification (*azu1*, *hemN*, *nnrU*, and *nnrS*) as NnrR targets (Meilhoc et al., 2010). In contrast to *B. diazoefficiens*, *E. meliloti* NnrR and FixK are part of two different regulatory cascades (reviewed in Cabrera et al., 2011).

NnrR orthologs are present in other organisms, such as NnrR of *R. sphaeroides* 2.4.1. In cells exposed to nitrosative stress, the *norCBQD* gene cluster, other genes encoding putative accessory proteins for the assembly of cNor as well as others involved in NO metabolism were identified (Arai et al., 2013). Similar to our findings for *B. diazoefficiens* NnrR, microarrays analysis of cells of a *P. aeruginosa* Dnr mutant cultured under anaerobic conditions revealed that the denitrification structural genes *nirS*, *norCB* and *nosR* as well as *hemN*, and genes encoding *c*-type cytochromes are controlled by this protein (Rodionov et al., 2005; Trunk et al., 2010). In the same way, DnrF from the marine bacterium *D. shibae* is required for the induction of the expression of *norCB*, *hemA* encoding the 5-aminolevulinate synthase, and a gene responsible for cytochrome *c* biosynthesis (Ebert et al., 2017).

We also carried out an in-depth characterization of *cycA*, one of the targets under the positive control of NnrR. By performing qRT-PCR and heme *c*-staining analyses, we were able to confirm the requirement of NnrR for the maximal expression of *cycA* in cells cultivated in anoxic denitrifying conditions. In addition to NnrR, the anoxic induction of *cycA* was also controlled by FixK_2_. However, *cycA* did not appear to be a target for FixK_2_ in previous microarray analyses of a *fixK*_2_ mutant grown microoxically (Mesa et al., 2008). We therefore postulate that anoxia might be required for FixK_2_-mediated control of *cycA* induction. In fact, similar heme *c*-staining levels of CycA were observed in the *fixK*_2_ mutant with respect to those in wild-type cells cultured either under microoxic or oxic conditions. However, the significant induction of CycA observed in the WT in response to anoxic denitrifying conditions in comparison to oxic or microoxic conditions was not observed in *fixK*_2_ mutant cells. The involvement of FixK_2_ on *cycA* induction under anoxic conditions was also confirmed at transcriptional level in qRT-PCR experiments. Moreover, we could also identify *cycA* as a direct target for FixK_2_ based on the FixK_2_-dependent *cycA* transcript detected in IVT activation experiments.

In previous studies, purified FixK_2_ protein has been shown to activate transcription in concert with *B. diazoefficiens* RNA polymerase from *napEDABC*, *nirK*, and *nosRZDFYLX* promoters, but not from the *norCBQD* promoter (Bueno et al., 2017; Torres et al., 2017), being NnrR the candidate to directly control *norCBQD* genes expression. In this work, we demonstrated that *nnrR* and its divergently oriented gene *nnrS* are two newly identified targets directly activated by FixK_2_. These results are in agreement with the dependency of both *nnrR* and *nnrS* induction on FixK_2_ (Mesa et al., 2003, 2008) and they also ratified that control of FixK_2_ on *norCBQD* genes is indirect via NnrR.

Gene regulation by NnrR could be direct (as proposed for *norCBQD*; Bueno et al., 2017), indirect (as reported for *nosRZDFYLX* and *nirK*; Torres et al., 2017) or with the involvement of another regulatory factor such as FixK_2_ (as suggested for *nirK*; Bueno et al., 2017). In principle, FixK_2_ and NnrR could also act together to regulate other targets identified that showed differential expression in the *nnrR* mutant. However, the presence of purified NnrR did not increase specific FixK_2_-mediated transcription from the *nirK* promoter in an IVT assay (Bueno et al., 2017) implying that the purified NnrR protein does not interact with FixK_2_ or it is not in the correct conformation to drive transcription. Further, IVT activation of target genes triggered by NnrR seemed to be recalcitrant (Bueno et al., 2017), therefore, at the moment, it is difficult to determine the most plausible mechanism involved in the NnrR-mediated control.

As mentioned above, CycA is a soluble cytochrome involved in anaerobic growth of *B. diazoefficiens* using nitrate or nitrite as final electron acceptor that suggests a role of this cytochrome in denitrification (Bott et al., 1995; Bueno et al., 2008). Specifically, it is involved in the electrons transfer from cytochrome *bc*_1_ to nitrite reductase (NirK) (Bueno et al., 2008). We have now reported, for the first time in *B. diazoefficiens*, the role of CycA in nitrous oxide reductase functioning, given the reduced N_2_OR activity observed in the *cycA* mutant compared to that of the WT. In addition to its involvement in the denitrification process, CycA plays a role in chemolithotrophic growth of *B. diazoefficiens* when thiosulfate is used as electron donor (Masuda et al., 2017). In this growth condition, electrons are preferentially channeled via cytochrome CycA to the *aa*_3_-type heme-copper cytochrome oxidase encoded by *coxBAFC* genes. Here, it is noteworthy that NirK, NosZ, and CoxB have copper (Cu) centers, particularly NosZ and CoxB contain Cu centers type A (Cu_A_) (Bühler et al., 2010). These observations let us to suggest that CycA might be responsible for electron transfer to Cu-containing proteins belonging to different respiratory pathways.

Importantly, the *cycA* mutant retained partial N_2_OR activity, which suggests that alternative electron donors might be also involved in electron transfer to Nos. This has been observed in other model denitrifying bacteria (reviewed in van Spanning, 2011; Torres et al., 2016) and the presence of alternative electron transfer routes was also proposed for chemolithoautotrophic growth as reported by Masuda et al. (2017). To identify other candidates as potential electron donors to Nos, we determined N_2_OR activity in *B. diazoefficiens* strains defective in the soluble cytochromes *c*_2_ encoded by bll2388 and CycS encoded by bll6062 (Mesa et al., 2008). However, as shown in Supplementary Figure S1, none of these mutants showed a clear negative effect on N_2_OR activity compared to wild-type activity levels. Thus, we propose the soluble cytochrome CycA is an important but not the only electron donor to the nitrous oxide reductase.

In summary, this work has contributed to increase the understanding of the regulation of denitrification in the model rhizobial denitrifier *B. diazoefficiens* by expanding the regulon of NnrR, one of the key proteins involved in the control of this process. The *cycA* gene, one of the identified targets, resulted also to be directly activated by the transcription factor FixK_2_. Moreover, we showed the direct link between FixK_2_ and *nnrR*, further highlighting the role of NnrR as an intermediary in the FixK_2_-mediated control of some target genes. Finally, we reveal an extra role for the soluble cytochrome CycA in the electron transfer pathway to Nos, adding a new player for nitrous oxide reduction in endosymbiotic soil bacteria.

## Data Availability

The microarray expression data are available in the NCBI Gene Expression Omnibus (GEO) database (http://www.ncbi.nlm.nih.gov/geo) under the GEO Series accession number GSE130684.

## Author Contributions

SM and MD conceived and designed the study. AJ-L, JC, EB, MT, and SS performed the experiments. AJ-L, MD, and SM analyzed the results and wrote the manuscript. AJ-L prepared the figures and tables. JC contributed to the set-up of some methodologies used in this study, the preparation of figures and tables, and the manuscript writing. EJB critically revised the manuscript. All authors read and approved the final version of the manuscript.

## Conflict of Interest Statement

The authors declare that the research was conducted in the absence of any commercial or financial relationships that could be construed as a potential conflict of interest.

## Supplementary Material

FIGURE S1 Nitrous oxide reductase (N_2_OR) activity in the absence and in the presence of succinate as electron donor determined in the WT and *cy*_2_, and *cycS* mutants. Activity is expressed in nmol N_2_O consumed per (mg prot)^–1^h^–1^. Cells were grown microoxically (2% O_2_) for 24 h in YEM medium supplemented with nitrate. A *nosZ* mutant was included as negative control. Shown are means with standard errors of a representative experiment assayed in triplicates.

TABLE S1 Strains and plasmids used in this study.

TABLE S2 Oligonucleotides used in this study.

TABLE S3 List of the 175 genes induced under anoxic denitrifying conditions and at the same time their expression is downregulated in the *nnrR* mutant in comparison to the WT, both cultivated in anoxic denitrifying conditions.

DATA SHEET S1 References cited in the Supplementary Material.

DATA SHEET S2 Full scans of the entire gels of the blots shown in Figure 3A **(A)**, and Figure 5B **(B)**. Specific details for each image are identical to those described in the corresponding legend to the figure. In **(B)**, the 50 kDa band detected in the WT and *cycA* mutant corresponds to the C-terminal truncated *B. diazoefficiens* NosZ protein as previously reported for NosZ from *P. denitrificans* (Felgate et al., 2012). A 50 kDa band was also present in the soluble fraction of the *nosZ* insertion mutant which produces a shorter 48.7 kDa polypeptide (deletion at the C-terminal end).
