# Supplementary figures and images for: Expanding the Regulon of the Bradyrhizobium diazoefficiens NnrR Transcription Factor: New Insights Into the Denitrification Pathway
Source: Front Microbiol. 2019 Aug 20;10:1926. doi: 10.3389/fmicb.2019.01926 (PMC6710368; doi:10.3389/fmicb.2019.01926)

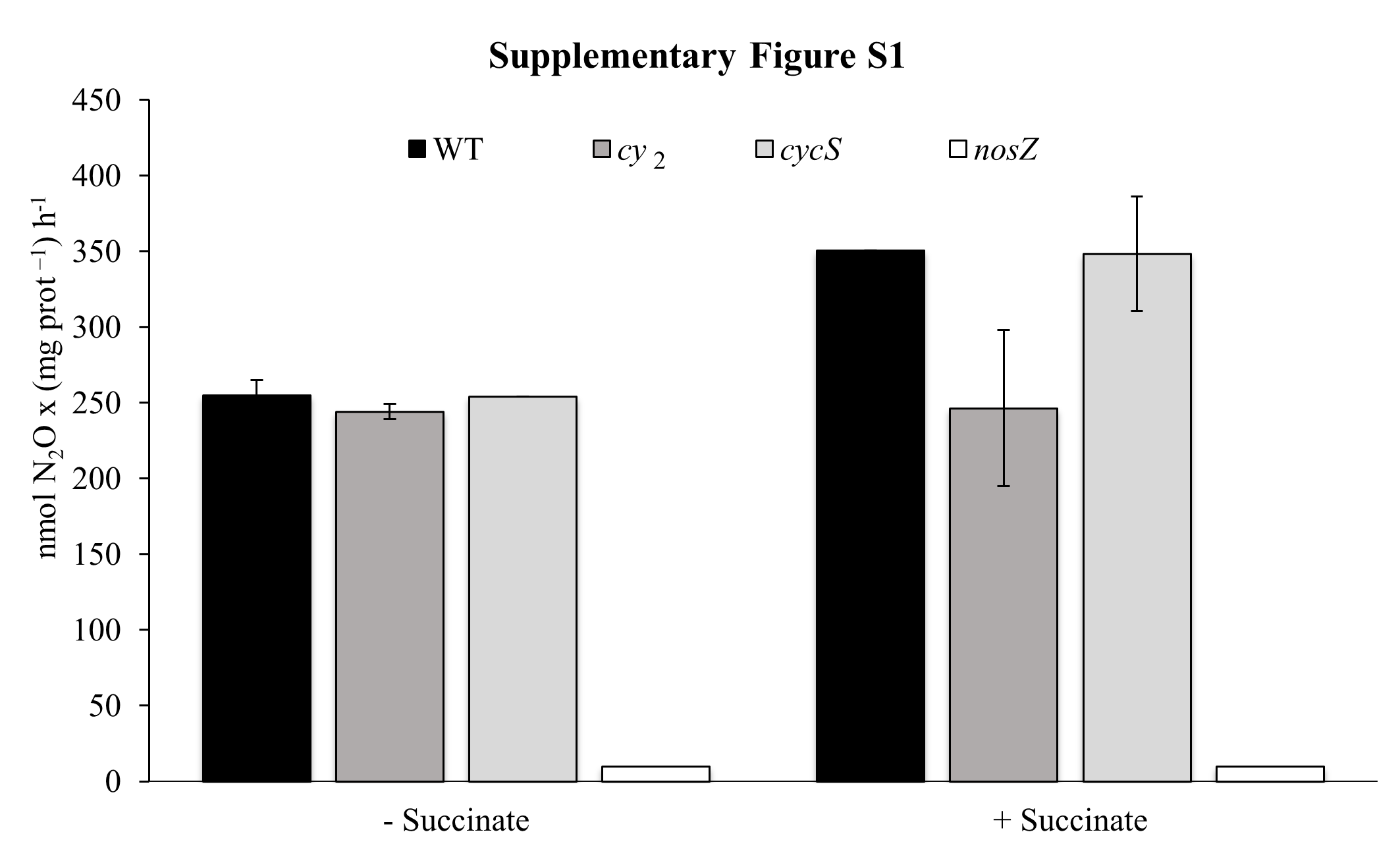

Supplement: FIGURE S1 — Nitrous oxide reductase (N2OR) activity in the absence and in the presence of succinate as electron donor determined in the WT and cy2, and cycS mutants. Activity is expressed in nmol N2O consumed per (mg prot)–1h–1. Cells were grown microoxically (2% O2) for 24 h in YEM medium supplemented with nitrate. A nosZ mutant was included as negative control. Shown are means with standard errors of a representative experiment assayed in triplicates. [file Image_1.TIF]
