# Supplementary material for: Expanding the Regulon of the Bradyrhizobium diazoefficiens NnrR Transcription Factor: New Insights Into the Denitrification Pathway
Source: Front Microbiol. 2019 Aug 20;10:1926. doi: 10.3389/fmicb.2019.01926 (PMC6710368; doi:10.3389/fmicb.2019.01926)
Supplement: TABLE S1 — Strains and plasmids used in this study. [file Table_1.DOCX]

**TABLE 1.** Strains and plasmids used in this study.

| Strains | Relevant description | Resistance | Source of reference |
| --- | --- | --- | --- |
| *E. coli* |  |  |  |
| BL21 | F^_^ *opmT* *hsdS*_B_(rB^_^ mB^_^) *gal dcm* (DE3) |  | Novagen Inc. |
| S17-1 | *thi,* *pro*, *recA*, *hsdR*, *hsdM*, RP4Tc::Mu, Km::Tn7 | Tp^r^ Sm^r^ Sp^r^ | Simon et al.,1983 |
| *B. diazoefficiens* |  |  |  |
| USDA 110 | Wild type | Cm^r^ | United States Department of Agriculture, Beltsville, MD, United States |
| 110*spc*4 | Wild type | Cm^r^ Sp^r^ | Regensburger and Hennecke, 1983 |
| 3447 | *cycA*::Tn5-*233* | Km^r^ Gm^r^ | Bott et al., 1995 |
| GRZ3035 | *nosZ*::Ω | Cm^r^ Sp^r^ Sm^r^ | Velasco et al., 2004 |
| 9043 | *fixK_2_*::Ω | Cm^r^ Sp^r^ Sm^r^ | Nellen-Anthamatten et al., 1998 |
| 8678 | Δ*nnrR*::*aphII* | Cm^r^ Sp^r^ Km^r^ | Mesa et al., 2003 |
| 8882 | Δ*cycS::aphII* | Cm^r^ Sp^r^ Km^r^ | Mesa et al., 2008 |
| 2003 | Δ*cy_2_* | Cm^r^ Sp^r^ | This work |
| Plasmids |  | | |
| pGEM-T Easy | *lacZ*, cloning vector | Ap^r^ | Promega |
| pK18*mobsacB* | Mobilizable pUC18 derivative, *mob,sacB* | Km^r^ | Schäfer et al., 1994 |
| pMB2000 | (pGEM-T easy) 5'-flanking region of *cy_2_* on a 614-bp PCR amplified fragment | Ap^r^ | This work |
| pMB2001 | (pGEM-T easy) 3'-flanking region of *cy_2_* on a 609-bp PCR amplified fragment | Ap^r^ | This work |
| pMB2002 | (pK18*mobsacB*) 615-bp *Bam*HI/*Eco*RI fragment from pMB2000 | Km^r^ | This work |
| pMB2003 | (pMB2002) 634-bp *Bam*HI/*Pst*I fragment from pMB2001 | Km^r^ | This work |
| pRJ9601 | [pBluescript SK(+)] *B. diazoefficiens rrn* promoter and *rrn* terminator on a 468-bp *Sac*I-*Sma*I fragment | Ap^r^ | Beck et al., 1997 |
| pRJ9519 | [pBluescript SK(+)] 308-bp *Bst*XI-*Kpn*I fragment containing the *B. diazoefficiens rrn* terminator cloned into the *Hin*cII and *Kpn*I sites | Ap^r^ | Beck et al., 1997 |
| pRJ8870 | (pRJ9519) 210-bp *Sac*II-*Xba*I fragment containing a second  *B. diazoefficiens rrn* terminator cloned into the *Sac*II and *Xba*I sites | Ap^r^ | Mesa et al., 2008 |
| pRJ8817 | (pRJ9519) *fixGHIS* promoter on a 524-bp *Xba*I-*Eco*RI fragment | Ap^r^ | Mesa et al., 2005 |
| pMB1400 | (pR8870) *nnrS-nnrR* intergenic region on a 258-bp *Bam*HI-*Eco*RI fragment | Ap^r^ | This work |
| pMB1401 | (pRJ9519) *cycA* promoter on a 289-bp *Hind*III-*Eco*RI fragment | Ap^r^ | This work |
| pRJ0004 | [pET-24c(+)] with a 701-bp *Nde*I/*Not*I fragment encoding C183S FixK_2_ His_6_ | Km^r^ | Bonnet et al., 2013 |
