# Supplementary material for: Expanding the Regulon of the Bradyrhizobium diazoefficiens NnrR Transcription Factor: New Insights Into the Denitrification Pathway
Source: Front Microbiol. 2019 Aug 20;10:1926. doi: 10.3389/fmicb.2019.01926 (PMC6710368; doi:10.3389/fmicb.2019.01926)
Supplement: TABLE S2 — Oligonucleotides used in this study. [file Table_2.DOCX]

**Supplementary Table S2**. Oligonucleotides used in this study.

| **Primer name** | **Gene** | **DNA sequence (5’ → 3’)** | **Source of reference** |
| --- | --- | --- | --- |
| **Mutagenesis** | | | |
| Amplification of the 5’-flanking region of *cy_2_* | | | |
| BLL1F | *cy_2_* | GCTCTAGAGCACTAGTCGGTCAGTCCCTTCTCCTTT | This work |
| BLL1R | *cy_2_* | CGGGATCCCGGCATGAGCACGAAGATCAGA | This work |
| Amplification of the 3’-flanking region of *cy_2_* | | | |
| BLL2F | *cy_2_* | CGGGATCCCGGCCAATGTCATCGCCTATCT | This work |
| BLL2R | *cy_2_* | CGGAATTCCGGATCATGCCCGATGTCGT | This work |
| **qRT-PCR** | | | |
| norC_3_for | *norC* | GCAGATGCCGCAGTTCAAC | Torres et al., 2017 |
| norC_3_rev | *norC* | TGATCGTGCTCACCCATTG | Torres et al., 2017 |
| nosR_qRT_PCR_F | *nosR* | ATGATCCAGGTGCGGCTGAAG | Torres et al., 2017 |
| nosR_qRT_PCR_R | *nosR* | CCGGCTGTGATGATTGTGTTCG | Torres et al., 2017 |
| cycA_for_1 | *cycA* | AACAAGAATTCCGGCATCAC | Torres et al., 2014 |
| cycA_rev_1 | *cycA* | TGATCTCGGTCTCGTTCTTG | Torres et al., 2014 |
| SigA-1069F | *sigA* | GAGATCATCGTCGAGGTGAAG | Lindemann et al., 2007 |
| SigA-1155R | *sigA* | GCGCTTGTTGATGTCGTAGA | Lindemann et al., 2007 |
| bll2388_for | *cy_2_* | GAATGTCATCGACCGCAAG | Torres et al., 2014 |
| bll2388_rev | *cy_2_* | TTGCATCAGAATAGGCGAAG | Torres et al., 2014 |
| rpoN1-qRT-for | *rpoN_1_* | TAACTTGCGCGAGTGCCTG | This work |
| rpoN1-qRT-rev | *rpoN_1_* | CTTGGCGAGGAGATCGAGATG | This work |
| **IVT template** | | | |
| cycA_1_for_IVT | *cycA* | ATAGAATTCCGCCAGCACTGCCTGGTC | This work |
| cycA_1_rev_IVT | *cycA* | CGCAAGCTTAAGGGTGCTCTTCGTCTCTTCC | This work |
| nnrR_6_for | *nnrR/nnrS* | ATAGGATCCAGCGGCCAGCCCGCAAAGTTG | This work |
| nnrR_6_rev | *nnrR/nnrS* | ATAGAATTCCCAGGGCTTCCGGCGTGACCC | This work |
