# Supplementary material for: Expanding the Regulon of the Bradyrhizobium diazoefficiens NnrR Transcription Factor: New Insights Into the Denitrification Pathway
Source: Front Microbiol. 2019 Aug 20;10:1926. doi: 10.3389/fmicb.2019.01926 (PMC6710368; doi:10.3389/fmicb.2019.01926)
Supplement: DATA SHEET S1 — References cited in the Supplementary Material. [file Data_Sheet_1.PDF]

Supplementary Data Sheet 1. References cited in the Supplementary Material.

Beck, C., Marty, R., Klausli, S., Hennecke, H., and Gottfert, M. (1997). Dissection of the transcription machinery for housekeeping genes of *Bradyrhizobium japonicum*. *J. Bacteriol.* 179, 364-369. doi: 10.1128/jb.179.2.364-369.1997

Bonnet, M., Kurz, M., Mesa, S., Briand, C., Hennecke, H., and Grutter, M.G. (2013). The structure of *Bradyrhizobium japonicum* transcription factor $FixK_2$ unveils sites of DNA binding and oxidation. *J. Biol. Chem.* 288, 14238-14246. doi: 10.1074/jbc.M113.465484

Bott, M., Thony-Meyer, L., Loferer, H., Rossbach, S., Tully, R.E., Keister, D., et al. (1995). *Bradyrhizobium japonicum* cytochrome c_{550} is required for nitrate respiration but not for symbiotic nitrogen fixation. *J. Bacteriol.* 177, 2214-2217. doi: 10.1111/j.1574-6968.2007.01034.x

Fernández, N., Cabrera, J.J, Varadarajan, A.R, Lutz, S., Ledermann, R., Roschitzki, B., et al. (2019) An integrated approach unveils new aspects of microoxia-mediated regulation in *B. diazoefficiens*. *Front. Microbiol.* 10:924. doi: 10.3389/fmicb.2019.00924

Kaneko, T., Nakamura, Y., Sato, S., Minamisawa, K., Uchiumi, T., Sasamoto, S., et al. (2002). Complete genomic sequence of nitrogen-fixing symbiotic bacterium *Bradyrhizobium japonicum* USDA110. *DNA Res.* 9, 189-197. doi: 10.1093/dnares/9.6.189

Lindemann, A., Moser, A., Pessi, G., Hauser, F., Friberg, M., Hennecke, H., et al. (2007). New target genes controlled by the *Bradyrhizobium japonicum* two-component regulatory system RegSR. *J. Bacteriol.* 189, 8928-8943. doi: 10.1128/JB.01088-07

Mesa, S., Bedmar, E.J., Chanfon, A., Hennecke, H., and Fischer, H.M. (2003). *Bradyrhizobium japonicum* NnrR, a denitrification regulator, expands the FixLJ-$FixK_2$ regulatory cascade. *J. Bacteriol.* 185, 3978-3982. doi: 10.1128/JB.185.13.3978-3982.2003

Mesa, S., Hauser, F., Friberg, M., Malaguti, E., Fischer, H.M., and Hennecke, H. (2008). Comprehensive assessment of the regulons controlled by the FixLJ-$FixK_2$-$FixK_1$ cascade in *Bradyrhizobium japonicum*. *J. Bacteriol.* 190, 6568-6579. doi: 10.1128/JB.00748-08

Mesa, S., Ucurum, Z., Hennecke, H., and Fischer, H.M. (2005). Transcription activation *in vitro* by the *Bradyrhizobium japonicum* regulatory protein $FixK_2$. *J. Bacteriol.* 187, 3329-3338. doi: 10.1128/JB.187.10.3329-3338.2005

Nellen-Anthamatten, D., Rossi, P., Preisig, O., Kullik, I., Babst, M., Fischer, H.M., et al. (1998). *Bradyrhizobium japonicum* $FixK_2$, a crucial distributor in the FixLJ-dependent regulatory cascade for control of genes inducible by low oxygen levels. *J. Bacteriol.* 180, 5251-5255

Regensburger, B., and Hennecke, H. (1983). RNA polymerase from *Rhizobium japonicum*. *Arch. Microbiol.* 135, 103-109. doi: 10.1007/BF00408017

Schäfer, A., Tauch, A., Jager, W., Kalinowski, J., Thierbach, G., and Puhler, A. (1994). Small mobilizable multi-purpose cloning vectors derived from the *Escherichia coli* plasmids pK18 and pK19: selection of defined deletions in the chromosome of *Corynebacterium glutamicum*. *Gene* 145, 69-73. doi: 10.1016/0378-1119(94)90324-7

Simon, R., Priefer, U., and Pühler, A. (1983). "Vector plasmids for *in-vivo* and *in-vitro* manipulations of gram-negative bacteria," in *Molecular Genetics of the Plant-Microbe Interactions*, ed. A. Pühler. (Berlin: Springer Verlag), 98-106

Torres, M.J., Argandoña, M., Vargas, C., Bedmar, E.J., Fischer, H.M., Mesa, S., et al. (2014). The global response regulator RegR controls expression of denitrification genes in *Bradyrhizobium japonicum*. *PLoS One* 9, e99011. doi: 10.1371/journal.pone.0099011

Torres, M.J., Bueno, E., Jiménez-Leiva, A., Cabrera, J.J., Bedmar, E.J., Mesa, S., et al. (2017). $FixK_2$ is the main transcriptional activator of *Bradyrhizobium diazoefficiens nosRZDYFLX* genes in response to low oxygen. *Front. Microbiol.* 8, 1621. doi: 10.3389/fmicb.2017.01621

Velasco, L., Mesa, S., Xu, C.A., Delgado, M.J., and Bedmar, E.J. (2004). Molecular characterization of *nosRZDFYLX* genes coding for denitrifying nitrous oxide reductase of *Bradyrhizobium japonicum*. *Antonie Van Leeuwenhoek* 85, 229-235. doi: 10.1023/B:ANTO.0000020156.42470.db
