# Supplementary material for: Expanding the Regulon of the Bradyrhizobium diazoefficiens NnrR Transcription Factor: New Insights Into the Denitrification Pathway
Source: Front Microbiol. 2019 Aug 20;10:1926. doi: 10.3389/fmicb.2019.01926 (PMC6710368; doi:10.3389/fmicb.2019.01926)

Supplementary Data Sheet 2: Full scans of the entire gels of the blots shown in Figures 3A (A), and 5B (B)

A **Profile of heme-stained soluble proteins from *B. diazoefficiens* WT, and *fixK₂*, *nnrR*, *cycA* mutants**

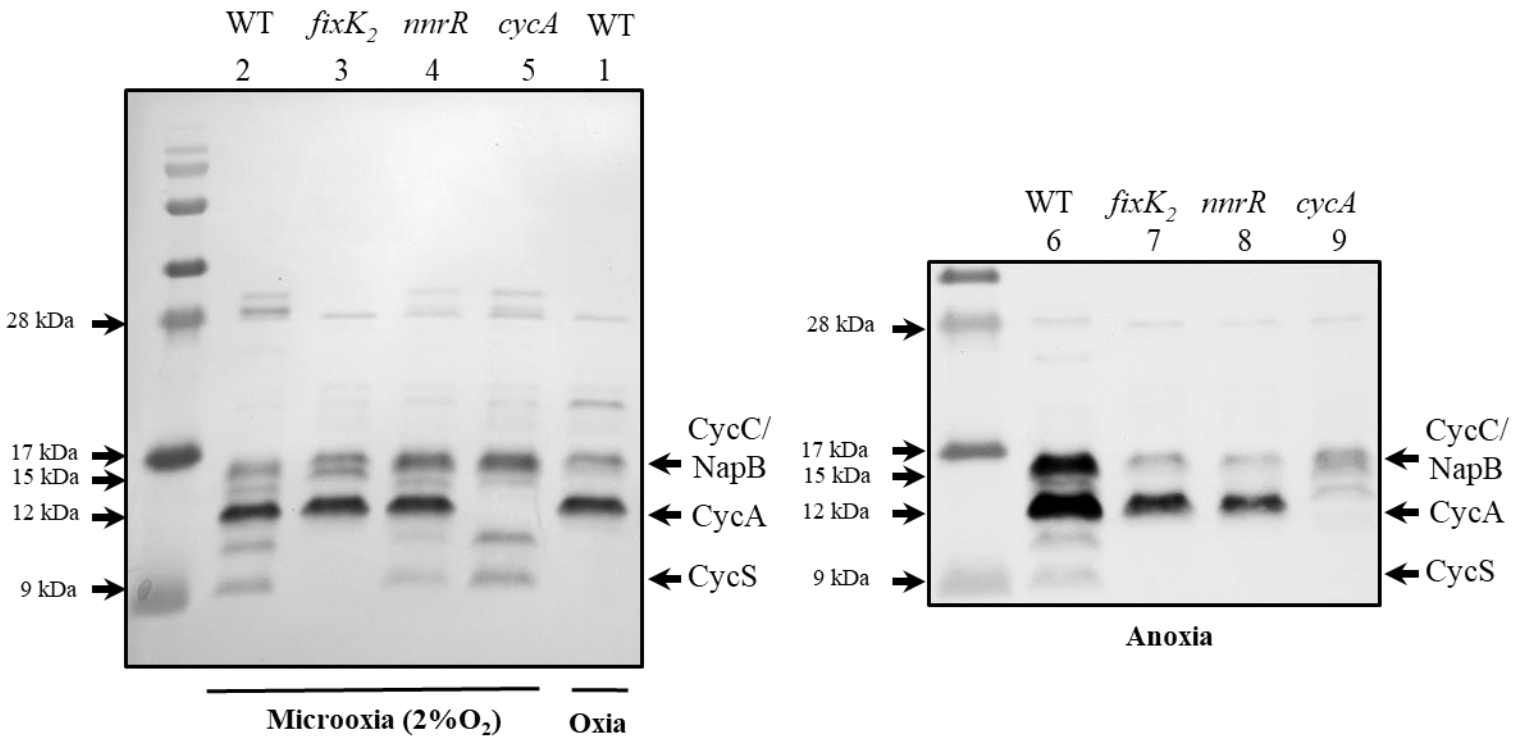

B **Inmunodetection of NosZ protein in the soluble fraction of the WT and *cycA* mutant**

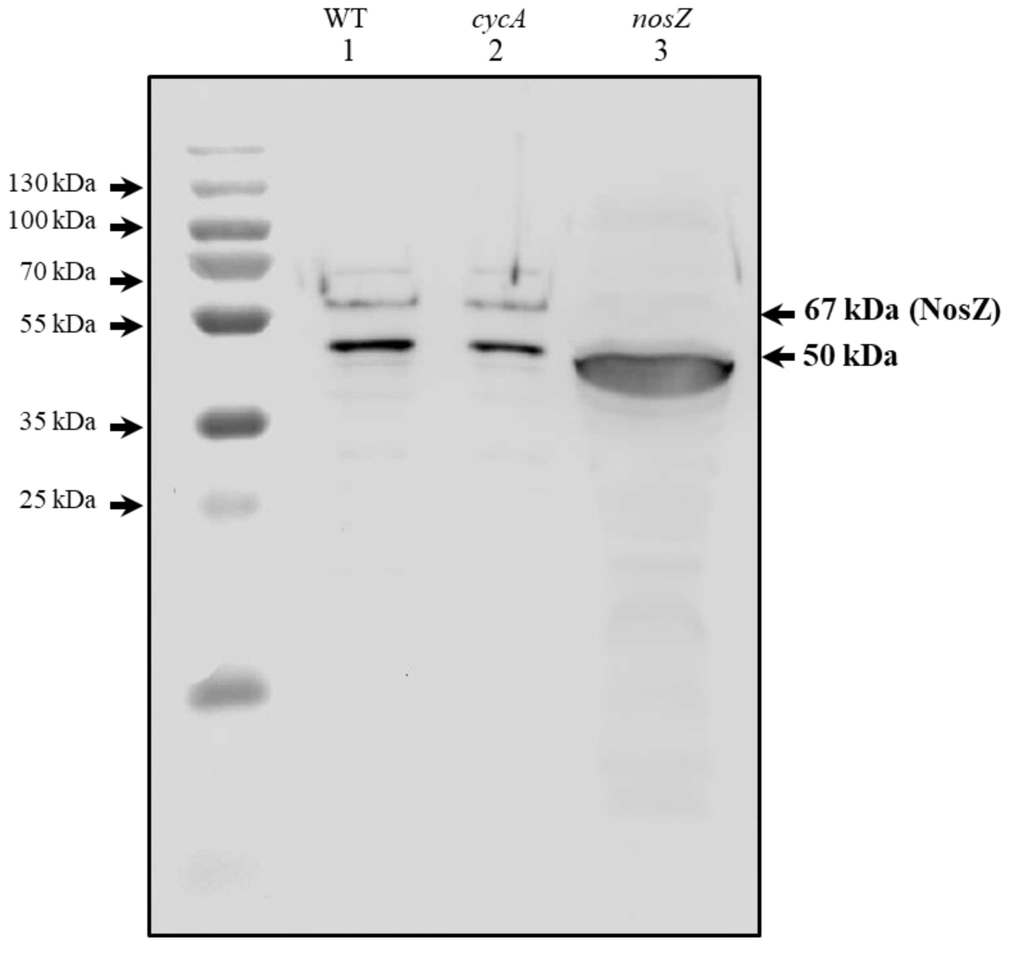

Supplement: DATA SHEET S2 — Full scans of the entire gels of the blots shown in Figure 3A (A), and Figure 5B (B). Specific details for each image are identical to those described in the corresponding legend to the figure. In (B), the 50 kDa band detected in the WT and cycA mutant corresponds to the C-terminal truncated B. diazoefficiens NosZ protein as previously reported for NosZ from P. denitrificans (Felgate et al., 2012). A 50 kDa band was also present in the soluble fraction of the nosZ insertion mutant which produces a shorter 48.7 kDa polypeptide (deletion at the C-terminal end). [file Data_Sheet_2.PDF]
